# Experiences of persons with multiple sclerosis with rehabilitation—a qualitative interview study

**DOI:** 10.1186/s12913-022-08150-8

**Published:** 2022-06-11

**Authors:** Donya Ghaidar, Anna Sippel, Karin Riemann-Lorenz, Christopher Kofahl, Rebecca Morrison, Ingo Kleiter, Stephan Schmidt, Christian Dettmers, Holger Schulz, Christoph Heesen

**Affiliations:** 1 Institute of Neuroimmunology and Multiple Sclerosis (INIMS), University Medical Center Hamburg-Eppendorf (UKE), Hamburg, Germany; 2 Institute of Medical Sociology, University Medical Center Hamburg-Eppendorf (UKE), Hamburg, Germany; 3 Independent Researcher, Berlin, Germany; 4 Marianne-Strauß-Klinik, Behandlungszentrum Kempfenhausen für Multiple Sklerose Kranke gGmbH, Berg, Germany; 5 Gesundheitszentrum St. Johannes Hospital, Bonn, Germany; 6 Kliniken Schmieder, Constance, Germany; 7 Department of Medical Psychology, University Medical Center Hamburg-Eppendorf (UKE), Hamburg, Germany; 8 Department of Neurology, University Medical Center Hamburg-Eppendorf (UKE), Hamburg, Germany

**Keywords:** Multiple sclerosis, Decision-making, Patient experiences, Rehabilitation, Qualitative study, Thematic analysis

## Abstract

**Background:**

Managing multiple sclerosis (MS) includes different treatment approaches. Rehabilitation is a key strategy in MS for improving functioning, activity and participation. As part of a larger study on overall patient experiences with different treatment approaches, this study aims to give an overview of different patients’ experiences and perspectives on inpatient rehabilitation in MS.

**Methods:**

We conducted problem-centered interviews in 50 persons with MS in Germany, of whom most had relapsing–remitting MS. We used the maximum variation sampling method during recruitment. Data were analyzed thematically.

**Results:**

As a result of the analysis, three major themes were identified: 1) factors contributing to the decision-making concerning rehabilitation, 2) experience with the rehabilitation setting, 3) benefits of rehabilitation treatments. The treating physicians’ attitude had a major impact on the decision to either opt for rehabilitation or not. Setting goals prior to rehabilitation was given a high priority. Exchanging experiences with other persons with MS presented a major benefit from rehabilitation while for some being separated from regular daily life resulted in a more ambiguous attitude ranging from appreciation of escaping daily hassles to substantial behavioral change management.

**Conclusion:**

Patients reported various experiences in the process of decision-making with regard to rehabilitation. Physicians´ advice, goal setting and the selection of the most suitable rehabilitation clinic were considered most relevant.

**Supplementary Information:**

The online version contains supplementary material available at 10.1186/s12913-022-08150-8.

## Background

Multiple sclerosis (MS) is an autoimmune inflammatory, demyelinating and degenerative disease of the central nervous system (CNS) with secondary axonal degeneration resulting in a wide variety of neurological symptoms [1]. At the beginning, 85% of persons with MS (pwMS) develop episodes with neurological disabilities, but full or partial recoveries, known as relapsing–remitting MS (RRMS). After 15–20 years, 60% to 70% of persons with RRMS have converted to the secondary-progressive course (SPMS). Appr. 15% are diagnosed with primary-progressive MS (PPMS), characterized by a slow, but steady increase of disability [2]. Physical and psychosocial impairment in MS may have enormous long-term effects on almost every aspect of daily living in persons with MS (pwMS) and their families. While the incidence of MS increases [3], epidemiological data indicate that the disease may take a more benign course at least in the 10–15 years after MS has been diagnosed [4, 5]. However, in 60–75% MS leads to substantial permanent disability [6].

Recently, the World Health Organization (WHO) has stressed that rehabilitation is of crucial relevance for medical management in the twenty-first century [7]. Societies face numerous challenges: aging populations, an increase in the prevalence of non-communicable and chronic conditions as well as health care costs and rising numbers of people recovering from injury and various illnesses. To meet these challenges, it will become more important to invest in rehabilitation which provides many opportunities: reducing the duration of hospitalization, increasing the functionality of individuals, keeping people in work, and increasing health and well-being. In 1980, and revised in 2001, the WHO has developed a framework to describe the consequences of diseases, which now are referred to as the international classification of functioning, disability, and health (ICF). The three major domains, namely impairments of body function and structure, activities of daily living and participation in the community have been defined for MS [8], enabling assessments and effectiveness of interventions. This is especially useful in the context of the holistic view in rehabilitation.

Although MS is not a curable disease, rehabilitation as a highly individualized process may help to improve and maintain the ability to perform basic daily functional activities, mobility, and participation in the society (e.g. occupation, communication and social integration) [9, 10]. By means of rehabilitation a large number of impairments and disabilities such as decreased mobility and fine motor skills, bladder and bowel dysfunction, disorders of speech and articulation as well as swallowing disorders and cognitive impairment may be addressed.

In terms of symptomatic treatment, multidisciplinary rehabilitation is the key approach in MS and studies have shown beneficial effects with regard to exercise and physical activity programs in particular [10, 11]. However, the evidence is limited largely due to limited rehabilitation access, limited research facilities and funding, diversity of symptoms, and complexity of study designs.

In Germany, rehabilitation programs financed by pension insurance providers are almost entirely offered on an inpatient basis. Therefore, in Germany, the term rehabilitation is predominantely understood as inpatient rehabilitation. Inpatient rehabilitation is a multimodal and multiprofessional training and problem-solving educational process to reduce impairment und support quality of life provided in a specialized clinic or a similar institution. The multidisciplinary team is led by physicians. Rehabilitation follows an application procedure mostly intiated by the treating physician. Although the financial costs of rehabilitation are covered by pension insurance or by health insurance, rehabilitation services are relatively rarely used by pwMS, especially in the early stages of disease [12]. A recent web-based survey with *n* = 590 pwMS indicated substantial barriers with regard to MS-rehabilitation, largely due to the lack of adequate information and missing support when applying for rehabilitation [12]. To our knowledge, personal attitudes towards and experiences of pwMS with rehabilitation in inpatient facilities in Germany have not yet been investigated, and especially, we are not aware of any qualitative research on this topic. Therefore, this qualitative study aims to explore: 1) the decision-making process of pwMS for or against rehabilitation in inpatient facilities, 2) experiences of pwMS within the rehabilitation setting, and 3) the experienced impact of rehabilitation on everyday life.

## Materials and methods

### Design

This interview study is part of the ‘PExMS’ (Patients’ experiences with multiple sclerosis) project [13], aiming to provide pwMS with an experiential information website as a decision aid for different therapeutic options such as disease-modifying therapies (DMTs), lifestyle adjustments, and rehabilitation. While the initial aim of the project was to collect patients’ experiences with DMTs, our study advisory board highlighted the importance of other treatment approaches (e.g. rehabilitation and lifestyle adjustments) for patients in managing MS. Therefore, we expanded our focus on patients’ experiences with different aspects of living with MS including different treatment approaches, such as rehabilitation, and included them in our newly created website (for more information on the PExMS project see [13, 14]). The research methods followed the recommendations for standardized qualitative research provided by international DIPEx (Database of Individual Patients' Experience of illness) association [15–17]. This collaboration of researchers and health professionals uses standardized qualitative research methods to understand patients’ experiences and tries to provide ‘balanced’ information from original interview data. However, PExMS was not a formal DIPEx project. The reporting of this study follows the recommendations of the consolidated criteria for reporting qualitative research (COREQ) [18].

### Participants

Participants for this qualitative interview study were recruited from clinics, rehabilitation centers and patient associations in Germany. As one of the major focuses of the project was to gather experiences of pwMS with DMTs [13] and RRMS is the most investigated course for which the intake of DMTs are approved for, we included persons with RRMS beyond age 18. Furthermore, the consent for the audio or video recording of the interview and accepting that the recordings would be displayed on a website were an inclusion criteria. Persons with PPMS, major cognitive deficits and/or poor German language skills were excluded from the qualitative interview study. We decided to keep patients in our study who came out as having SPMS during the interview process, since SPMS follows an initial RRMS course and the uncertain period in the transition from RRMS to SPMS can lead to difficulties in reliable distinction between RRMS and SPMS [19]. Our questions then referred to their experiences when having RRMS. We recruited pwMS via newsletters of the MS day hospital at the University Medical Center Hamburg-Eppendorf and during medical appointments at two rehabilitation centers in Germany using the maximum variation sampling method [20]. The aims were to gather patients’ experiences as broadly as possible and to obtain an adequate and appropriate sample with regard to different therapeutic approaches (rehabilitation, alternative medicine, lifestyle adjustments and especially DMTs), ages, sex and education levels. Also, we were interested on pwMS who decided not to chose a DMT or other therapeutic approaches. After the first 30 interviews, we constantly checked the generated data for the intended variability of experiences in relation to the criteria previously mentioned. Then we further recruited and searched either for more people with experiences with certain therapies or for certain age groups and sex.

### Data collection

The study was approved by the ethics committee of the Hamburg Chamber of Physicians (approval number: PV5770). Written informed consent was obtained by all participants.

Interviews using a problem-centered interview guide (Additional file 1) were collected from March 2018 to May 2020 including questions on pwMS’ experiences with diagnosis of MS, everyday life and different approaches of how to manage the disease, such as DMTs, lifestyle measures and rehabilitation (Appendix 1). The interview guide was mutually developed with an expert panel on qualitative methods in the clinical research group of the Institute of Neuroimmunology and Multiple Sclerosis (INIMS), and our advisory panel consisting of representatives of pwMS, researchers and neurologists and followed Witzel’s guidelines [21] for interview questions. The interview guide was pre-tested with five pwMS to ensure appropriateness of length and clarity. As no further adjustment seemed necessary, the interview guide and the five interviews were fully integrated in our study. A female research associate with a master’s degree in health science (AS) and experienced in qualitative research performed the interviews. These were audio- and videotaped and the audio tracks transcribed for the analyses. Interviews lasted between 20 and 97 min (mean 45.6 min) and were performed at a location preferred by the participants (e.g., clinic, home, workplace). The pwMS received an incentive of 20 €.

### Data analysis

A thematic analysis according to the six steps of Braun and Clarke [22] was applied to all transcripts using the qualitative data analysis software MAXQDA Analytics Pro 2018. We followed the realist approach and thereby reported patients’ experiences and meanings being evident in the data [23, 24]. Firstly, two of the authors (DG and AS) re-read the transcripts. Secondly, they created first ideas as initial codes associated with extracts of data. Thirdly, these different codes were grouped into potential themes, which captured common, recurring patterns across the dataset [22]. As a fourth step, the main themes were refined: I) the collated extracts were reviewed for each theme; II) then each theme was assessed from the viewpoint of a potentially coherent emerging pattern; III) overlaps between the themes were eliminated; IV) afterwards the thematic map was examined for discrepancies by peer discussion and by consideration of the current state of research to reflect the meaning evident in the data as a whole. Fifthly, names of each theme and sub-theme were refined to express the essence of each identified theme. Finally, a report on the results of the thematic analysis was written (step six). Each theme in this article is represented by quotations, which were translated from German into English by a translator. For clarification from how many and from which different persons the quotes were derived, the interviewees are represented by numbers (e.g. pwMS 5). In the following, for the ease of readability, the term rehabilitation is used for inpatient rehabilitation.

## Results

In total, we interviewed 50 pwMS (35 females) between 21 and 61 years old. Those who had experiences with rehabilitation (*N* = 27), had a longer duration of illness and higher disability (mean Patient Determined Disease Steps = 3.6) (Table 1).

**Table 1:** Demographics and MS-related characteristics of participants

Characteristics	N (%)	N (%)	N (%)
	Total (*n* = 50)	Experience with inpatient rehabilitation (*n* = 27)	No experience with inpatient rehabilitation (*n* = 23)
Sex			
Females	35 (70)	19 (54)	16 (46)
Males	15 (30)	8 (53)	7 (47)
Age in years (mean, range)	44.4 (21 – 61)	48.9 (32 – 61)	39.0 (21 – 57)
Highest professional qualification			
No professional qualification	3 (6)	0 (0)	3 (13)
Vocational education	27 (54)	17 (63)	10 (43)
Academic degree	20 (40)	10 (37)	10 (43)
MS type			
RRMS	44 (88)	21 (48)	23 (52)
SPMS	6 (12)	6 (100)	0 (0)
Duration of MS since diagnosis (mean, range)	13.4 (2 – 33)	15.0 (4 – 28)	11.6 (2 – 33)
Patient determined disease steps (PDDS) (mean, range)	2.7 (0 – 7)	3.6 (1 – 7)	1.8 (0 – 5)

*RRMS* Relapsing–remitting MS, *SPMS* Secondary-progressive MS, *PDDS* Range from 0 (normal) to 8 (bedridden)

**Fig. 1:**
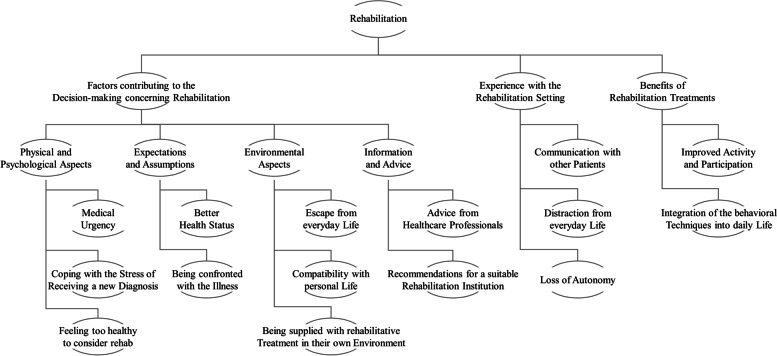
Thematic map of themes and sub-themes of positive and negative pwMS’ attitudes and experiences with inpatient rehabilitation

Data yielded three major themes with regard to experiencing rehabilitation: 1) factors contributing to the decision-making concerning rehabilitation; 2) experience with the rehabilitation setting; and 3) benefits of rehabilitation treatments (Fig. 1).

### Factors contributing to the decision-making concerning rehabilitation

The first theme describes which factors encouraged pwMS to decide for or against rehabilitation in inpatient facilities.

#### Physical and psychological aspects

Seventeen patients reported that their acute health status had required treatment in an inpatient rehabilitation facility in the past. Most patients decided upon rehabilitation, as a matter of medical urgency, shortly after the diagnosis had been made or as a consequence of a relapse with incomplete remission in the course of follow-up treatment in order to fully recover or at least alleviate the remaining symptoms.

“The decision process for the first rehab stay […] happened after diagnosis, or after that first relapse. At that point, I couldn’t decide much for myself. It was just obvious that I required some rehab to help improve my walking.” (pwMS 25).

A few patients decided upon a rehabilitation stay after the diagnosis had been made to gain a better understanding of the disease and to cope with the stress and anxiety evolving because of having been diagnosed with MS.

“This means that the rehab clinic kind of takes you in hand right at the start, because you’ve just received a new diagnosis, everything is different, so much has changed, and you are really frightened […]. I was in a wonderful rehab clinic, and it had a special program, a so-called Training Program, which was like being a beginner at school again.” (pwMS 28).

One patient felt completely left alone after the diagnosis of MS had been made in the hospital.

“And then I was discharged [from hospital], with no advice on what I should do next. All I had was the information on those flyers. The sum of my knowledge when discharged. And then rehab was mentioned. That was at diagnosis time.” (pwMS 42).

A common factor for a decision against a rehabilitation program was that some pwMS felt too healthy to consider rehabilitation.

“Because I always felt fit enough already, I did sport, and the limitations imposed on my body weren’t so huge.” (pwMS 40).

#### Expectations and assumptions

When deciding for or against inpatient rehabilitation, pwMS were influenced by their expectations and assumptions regarding the inpatient rehabilitation.

Many patients who decided in favor of rehabilitation expected to gain a better health status, e.g. to regain “normal gait”. Accordingly, some patients emphasized the importance of defining specific goals prior to rehabilitation and of applying rehabilitative techniques in daily living to alleviate symptoms and maintain the achievements obtained by inpatient rehabilitation.

“Simply attending rehab with an expectation that then I’ll be better, isn’t workable […] I had set myself the goal of finding out what I could do to help my walking […] most of all for when I was back home, to really consider the question, what can I integrate into my everyday life? It’s no good thinking that a visit to rehab is all it takes.” (pwMS 22).

Some pwMS explained that participating in rehabilitation gave them the opportunity to fully focus on physical training, thereby increasing the chances of a beneficial effect substantially.

“I really put that into practice because I believe it is more effective to do something every day than to go to physio once or twice a week, that this is the way to stay fully charged […]. That is what really swung it for me for residential rehab, rather than doing it from home.” (pwMS 7).

The thought of being confronted with the disease in all its facets especially shortly after the diagnosis was a factor in deciding against participating in inpatient rehabilitation. Some participants decided against rehabilitation expecting to be negatively affected when meeting other pwMS supposed to be more disabled than themselves.

“I actually never did rehab. I was always put off by the thought I would see people there who were worse off than me and that this wouldn’t be good for me. That is what has stopped me from going thus far.” (pwMS 2).

“Well, you know, going to rehab straight away after that first relapse, seeing other MS patients, the full spectrum from those who are like me, where it’s not visible, to those who are in wheelchairs, that can really pack quite a punch to begin with, it’s quite something to process mentally.” (pwMS 22).

#### Environmental aspect

The professional and private environments of pwMS, such as the workplace and private surroundings, yielded conflicting reactions by either playing a facilitating role or being a barrier to some patients.

Many participants wished to escape from everyday life. Some pwMS wished to obtain a break from their everyday life by finding time to relax on the one hand and to fully concentrate on MS on the other. Subsequently, they wished to be able to focus less on the disease once they have returned to their home.

“It feels good, simply having time away from work, from family, from everyday life, and immersing yourself there where the subject matter is the disease […]. For it’s not through my illness that I want to live. I am living my life and this darned disease is a companion. But I do not want my everyday life to be defined by the disease.” (pwMS 26).

Others described that incompatibility of inpatient rehabilitation with their personal lives was an important factor to decide against it. One mother explained that she had never been able to realize a longer stay away from her home and children. Here the decision against a rehabilitation strongly points to a gender issue.

“No, I’ve never been away on rehab. […] I’d say that’s definitely to do with my husband who said: Well now, what am I supposed to do with the children, and so on and so forth, and somehow it never came to be.” (pwMS 8).

A few pwMS decided against rehabilitation, because they had the opportunity to obtain adequate treatment in their own environment, emphasizing the wish to reconcile rehabilitation and private lives thereby increasing the chance for long-term results.

“After the hospital I actually had a really great physiotherapist and […] both my general practitioner and neurologist were people I trusted very much and I didn’t want to leave [these familiar surroundings]. I had a lot of thinking to do and for me the familiar setting was where I needed to do this.” (pwMS 48).

A possible alternative for pwMS who decided against inpatient rehabilitation would be an outpatient setting such as a day unit rehabilitation. Some participants felt that a specialized day unit was very much like an inpatient rehabilitation and therefore didn´t feel the need to apply for an inpatient rehabilitation.

“When I needed help, or when I noticed that I needed some more support, I was sure to go to the clinic. That is ultimately a lot like rehab anyway. You are there and cared for and have the best of therapists.” (pwMS 11).

#### Information and advice

PwMS also stressed the importance of communicating with their treating physician with regard to the decision to participate in inpatient rehabilitation. Some pwMS followed the physician’s advice on going to rehabilitation. One interviewee described receiving positive pressure from the treating neurologist to apply for rehabilitation. The citation indicates that patients in the beginnings of a negative spiral may require external guidance to exit this phase.

“The decision for rehab, yes, the first time that was on the advice of the doctors here, the second time there was a little push from the neurologist […]. It’s possible that this was during a phase when I was teetering on the edge a little and she could see that.” (pwMS 5).

Other pwMS demonstrated a more active approach by presenting their wish to participate in inpatient rehabilitation when seeing their treating physicians. These pwMS aimed to actively improve their state of health realizing that this objective could not be achieved along with the daily working load, and as a result, the neurologist reacted by recommending rehabilitation. The ensuing citation nicely illustrates the concept of enabling adaptation as the main aspect of rehabilitation.

“I knew that rehab away might be a possibility. And then I spoke to my neurologist about it and asked him: Is there anything we can do to stabilize my state of health, because I’m just not managing to do this in day-to-day life. […] And then he recommended it to me, and said, yes, this is something we can do.” (pwMS 22).

One pwMS reported that the physician disagreed with their proposal to apply for rehabilitation, claiming that the patient´s health status was too good. This attitude either points to a limited understanding of what can be achieved by rehabilitation or a recollection of bad experiences when these more educational objectives had not been met in rehabilitation.

“I was having issues with my walking […]. And so I asked my neurologist, I said, before it gets worse wouldn’t rehab be an option now, to prevent that? And I was told: No, you are too well for that.” (pwMS 50).

In Germany, according to the code of social law (§8 SGB IX), each patient in principle has the right to decide upon a certain inpatient rehabilitation facility or outpatient rehabilitation treatment best suited for their individual needs.

“I attend an MS group with regular meet-ups […] and lots of people there had already been and were really enthusiastic about [rehab residencies]. And that’s when I thought, right, I’m going to do it, too.” (pwMS 21).

“You chat about it with people and I also heard: […] “I’ve been in other clinics that were better” and then I researched a bit on the internet to find out what a clinic actually is and what they offer.” (pwMS 22).

However, only a few pwMS actively searched for information and recommendations for a suitable rehabilitation facility. The vast majority of pwMS agreed to the recommendation provided by the health insurance. Additional sources of information were other patients’ experience with rehabilitation either shared by pwMS in patient support groups or on the internet.

### Experience with the rehabilitation setting

The second theme is characterized by pwMS’ overall positive or negative experiences with inpatient rehabilitation.

#### Communication with other patients

Most participants found the personal contact with other pwMS in a rehabilitation facility particularly valuable, especially from a psychological point of view. PwMS described how helpful it was to gain a better understanding of the disease and learn about the spectrum of MS manifestations.

“Yes, it’s a place where you can discuss things with like-minded people. It’s where you get to know the various forms of MS. […]. And it’s also where you discover that MS does not automatically mean a wheelchair and full-time care, and psychologically, that is really important.” (pwMS 5).

Conversations with other pwMS were described as very helpful not only to cope with MS but also with regard to other life issues.

“To be honest, your fellow patients are the best therapists. Because we talk a lot to each other, privately, too, not only about the disease, but about everything, for everyday problems still exist for us, too. And that is the best therapy of all.” (pwMS 30).

#### Distraction from everyday life

As previously described in the context of influencing factors in the decision-making, escape from everyday life was indeed experienced by pwMS. Some pwMS even reported that the stay in a rehabilitation facility almost felt like a holiday. Moreover, they could fully focus on the treatment of their illness with the help of a multidisciplinary team without being distracted by their daily workload and issues.

“What I also found really great was simply switching off from the world, so that dream holiday that you always longed to have can actually be found at rehab, and it was a really lovely combination of medical input, sport, therapies of every stripe, shape and hue. […] You are away from home and those constant thoughts of tomorrow I have to go back to work, I must do the laundry, when am I going to cook […] instead you simply switch off and truly leave the everyday behind you.” (pwMS 10).

There were also pwMS with a more skeptical view on rehabilitation since the almost ideal circumstances were regarded unrealistic when compared to real life. One participant underlined the somewhat artificial setting of inpatient rehabilitation using the image of being under a “bell-jar.” However, these pwMS rather enjoyed these effects of rehabilitation to be able to calm down.

“The thing is, being at rehab for me is a little like being in a bell-jar, for these circumstances are not those of my everyday life. For example, I can get up and turn my attention immediately to the rehab task at hand […] and that is not real life. […] Everyday life is 1000 times more difficult for me than when I can restore away at rehab and […] move a little in the swimming pool or whatever. That is not the major challenge for me. Rather it is that peacefulness you experience at rehab.” (pwMS 35).

“Personally, I like being an inpatient for rehab. And I’ve had the good fortune of being in a really excellent clinic. You feel like you are in a bell-jar. Personally, I felt so cared-for there; the people there were great.” (pwMS 40).

#### Loss of autonomy

Five interviewees voiced negative impressions with regard to their stay in rehabilitation facilities, expressing feelings of lost autonomy and annoyance at having to act under orders.

“Well, at rehab I also heard a lot of lectures about what you should and shouldn’t eat, but I think for me, the disease already decides so much in my life. So I really don’t feel like being told in addition what I should eat or shouldn’t eat, or when I should be doing sport. That at least I want to decide myself.” (pwMS 10).

One pwMS participating in a psychosomatic rehabilitation clinic felt too tightly controlled and pressed into a scheme to function.

“It’s pretty tightly controlled: […] you get given numbers telling you where you have to sit at lunchtime and then that’s checked up on. […] Then in the evening, doors are locked, you can’t go out after 10 pm.” (pwMS 39).

Another pwMS perceived out-patient rehabilitation as a good alternative to the inpatient setting allowing more time to organize for themselves.

“Within the framework of rehab I always found the external control a real trial and burden and that’s why out-patient rehab was a really good option for me.” (pwMS 3).

### Benefits of rehabilitation treatments

Inpatient rehabilitation was considered to exert positive effects on their overall well-being, both mentally and physically by pwMS.

#### Improved activity and participation

In accordance with their expectations and assumptions, many pwMS reported physical benefits. More specifically, pwMS experienced an improvement of balance, spasticity and gait, sometimes even to an extent that their level of activity prior to the relapse or worsening of MS was regained.

“I attended […] medical rehab, […] I had sport therapy there and so on … and I entirely regained my ability to walk.” (pwMS 1).

Twenty-six pwMS felt mentally and physically empowered to take up new challenges.

“Oh yes, simply doing something at last, doing a bit of sport again. […] and then eventually going to bed with that feeling of satisfaction at what I’d achieved that day, for body and soul, and also in terms of friendship and general well-being.” (pwMS 17).

Complementary to being challenged, relaxation and stress management and just letting things flow were considered important aspects of improving well-being. By doing so, pwMS felt prepared to face another year of workload. In fact, many pwMS described participation in a rehabilitation program once a year as a kind of “refueling “, enabling them to carry on with their daily living for the next year.

“If I didn’t have rehabilitation in the year, I am quite sure that I would no longer be able to work on account of the fatigue, the exhaustion syndrome, because at rehabilitation I can let myself go entirely, can truly relax, and refuel the energy-tank, and thus be ready to more or less make it through another year of work.” (pwMS 25).

#### Integration of behavioral techniques into daily life

Participants reported how they applied newly learned techniques derived from balance training, stress management seminars, psychotherapy, mental exercise or nutritional counselling in their everyday life. They described how these measures helped them cope and better engage with some of the difficulties in daily life related to MS.

“When brushing my teeth, alternating from the right to the left leg, just that bit of movement. I learned that at rehab, and I do it. These are the little things that I have built into my everyday now.” (pwMS 25).

“And in the clinic I learned about occupational therapy […] for everyday life. Things like how to become more adept with the wheelchair; how to empty the washing machine whilst sitting in a wheelchair, how to hang out washing, how to cook meals, all those kinds of things.” (pwMS 37).

Some pwMS reported that rehabilitation even evoked fundamental changes of perspective with regard to MS and their lives almost as “flipping a switch”.

“I stayed at a rehab clinic this year and was particularly eager to discuss nutrition and I had lots of conversations with an assistant nutritional therapist who helped me so much there, who provided me with an incredible number of tips, and that was really like the flicking on of a switch for me.” (pwMS 46).

“The time at rehab helped me not only in terms of the disease itself, but also as regards my living environment as a whole, so my perspective on work, on certain personal stories, on problems really changed.” (pwMS 23).

Many pwMS became aware of the fact that in order to maintain or even increase treatment effects, exercise and relaxation techniques need to be applied continuously in their private surroundings.

## Discussion

To date, only very few attempts have been made to sample pwMS’ experiences with rehabilitation in a systematic way. This study addressed patients´ experiences with rehabilitation in MS demonstrating that the process of decision-making, goal setting and the selection of the most suitable rehabilitation facility for the individual needs were of critical importance.

When identifying the influencing factors in the process of decision-making for or against inpatient rehabilitation, the four subthemes ‘physical and psychological aspects’, ‘expectations and assumptions’, ‘environmental aspects’ and ‘information and advice’ emerged. This is in line with the findings of a qualitative study in Norway by Helland et al. [25]. Here, information about MS and rehabilitation were major determinants for rehabilitation use. Giesler et al. also underlined that in Germany, information about rehabilitation content was a relevant facilitator in the decision for rehabilitation and the lack of a transparent access process through insurances a barrier [12].

The decision for rehabilitation appeared to be easy in the context of a recent diagnosis of MS or a relapse with incomplete remission and the resulting physical impairment. The psychological consequences following the diagnosis of MS can be devastating, and consequently depression is frequent in pwMS [26]. In some pwMS, the main driving force in applying for rehabilitation treatment was linked to the perspective of being able to re-orientate and cope with the emotional distress of the diagnosis. This is especially relevant as participants stressed the relevance of early rehabilitation soon after diagnosis largely for educational purposes. However, in the case where psychological, educational, or neuropsychiatric aspects dominate, the regular neurological rehabilitation unit might not be able to meet these expectations. An earlier survey of 183 German neuro-rehabilitation units has shown that the expertise in these areas might not be sufficient enough in common neurological rehabilitation clinics in Germany [27]. In fact, as has recently been outlined in the MS care unit concept, a multitude of expertise is required, which might be difficult to meet in a general neurological rehabilitation clinic [28]. However, cohort data indicate that specialized programs for recently diagnosed pwMS are feasible and may especially increase mental quality of life [29].

The study results emphasize the critical importance of the treating physician´s attitude and communication regarding rehabilitation in the process of decision-making, as also pointed-out in the qualitative study by Helland [25]. In fact, there are a couple of specific barriers for the rehabilitation use in Germany. Although physicians play a central role in the highly formalized submission process to rehabilitation, they are only partially paid for this service. Moreover, rehabilitation is represented in the medical education only as a side line and most of the practicing neurologists have no experience of rehabilitation and what it might facilitate. Furthermore, rehabilitation clinics need to fulfil rigorous formal requirements for the paying institutions, and they need to cover a diversity of neurological conditions with different severity stages, thereby possibly not meeting the individual needs of patients.

In the process of deciding which inpatient rehabilitation facility might be most suitable for the individual patient, some pwMS sought advice from their social environment or information from other pwMS, which is in line with previous findings [30, 31]. Peer support and shared patient experiences have been highlighted as an unmet need in MS before [32], but the evidence for benefits of shared patient experiences effects in MS is ambiguous [33]. Moreover, some pwMS in our study expressed their concern to be confronted with other pwMS, which is also in accordance with the findings of Helland et al. [25]. Interviewees were particularly scared to meet pwMS with more pronounced neurological deficits revealing the full spectrum of potential neurological deficits caused by MS. The fact that only a small group of pwMS in our sample mentioned this concern, may be due to a selection bias since the interviewees participating in this study might represent pwMS, who are more extravert as demonstrated by the fact that they agreed to be interviewed and have parts of their video clips displayed on the internet. Most of our participants felt that communication with other pwMS at the rehabilitation clinic was a very important part of their treatment and emphasized the exchange with other MS patients as particularly positive.

Interviewees in our study expected to improve their health status during a rehabilitation stay. In accordance with recent work, they clarified that personally meaningful and specific goal setting prior to rehabilitation may help to increase motivation and to accept a possibly incomplete recovery [34]. Playford (2019) outlined the many facets of goal setting as building empathy, creating a contract, identifying priorities, summarizing the conversation, articulating the goal, defining actions, building coping plans and then reviewing [35]. However, the evidence on the impact of goal setting for successful rehabilitation is not strong [36]. Transferring of what has been achieved during rehabilitation was a major goal reported in the interviews. We conclude that more work on the relevance of goal setting for sustainability of rehabilitation effects is warranted.

Inpatient rehabilitation also offers the opportunity to escape from everyday life which one pwMS described as “dream holiday”, which was also a finding of the study by Helland [25]. For patients with a tendency to overcompensate for deficits, this escape scenario might be a particular challenge.

A few pwMS felt that inpatient rehabilitation was incompatible with their personal life. Of note, this argument was frequent in female pwMS, who felt unable to relinquish their family duties for a longer period. With regard to rehabilitation, pwMS tend to accept an inpatient setting more easily if their work, family, and social life is less affected [37].

Patients experienced the rehabilitation setting in different ways: Some revealed emotions such as feeling protected and cared for with the ability to fully concentrate on themselves and the disease and to “fuel the tank for another round”. Others felt separated from their private surroundings as well as put into an artificial setting not transferable to their daily life. Sometimes the holistic approach was negatively perceived as pwMS felt overwhelmed by management concepts touching all aspects of life. As mentioned above, setting meaningful goals might help to modify this ambiguity without fully resolving it.

With regard to possible achievements of rehabilitation, there were also conflicting results: Some pwMS felt that the mere escape from their daily duties was an achievement, whereas others acquired new behavioral techniques which could be transferred into their daily living, and still others reported that rehabilitation was a turning-point in their treatment history of MS. Of note, psychological achievements of rehabilitation were mentioned most frequently, although they may get lost within months as we recently have shown in a larger controlled multicenter study on a metacognitive intervention [38].

### Strengths and limitations

The significance of the data presented here may be limited by the fact that the data are derived from a cohort of pwMS with only mild to moderate disability, showing an interest in sharing their experiences by video files on the internet. Hence, we might have collected a non-representative sample of pwMS characterized by higher openness and extraversion than the “average” pwMS. Only about half of the interviewed patients really had experience with rehabilitation. On the one hand, this can be seen as a limitation as they may represent a subgroup who have managed to cope with the disease and are rather skilled with regard to rehabilitation. On the other hand, our study sample enabled us to gain insight into reasons for deciding against rehabilitation. However, the aspects that were discussed also cover the needs of pwMS in transition to or within a progressive phase [19]. Experiences of the six patients with SPMS did not differ qualitatively. Nevertheless, experiences of patients with advanced SPMS or PPMS were excluded from the study and conclusions can only be drawn for RRMS. The researcher who conducted the interviews (AS), and the researchers who analyzed the data (AS, DG), are female. They are educated in health science and medicine and are not personally affected by MS, which could have influenced the relationship to the pwMS and the richness of the outcome in both directions, negatively and positively. Finally, these findings apply to experiences with the German rehabilitation setting and therefore are only partially generalizable for rehabilitation in an international perspective. However, the detailed description of the research process and the study participants enable the reader to assess whether the findings are transferable to other settings [39, 40].

## Conclusion

In summary, this study indicates that pwMS may lack information about how to gain access to inpatient rehabilitation. They stressed the relevance for early rehabilitation soon after diagnosis for educational purposes. The input from other patients was considered important but ambivalent. The setting and structure of rehabilitation was mostly looked at as protective, but sometimes also as too rigid. A maintenance concept after rehabilitation was a major concern of most participants.

## Supplementary Information

**Additional file 1. **Interview guide.

## Data Availability

The generated and analyzed data are not publicly available due to privacy issues but can be obtained from the corresponding author upon reasonable request.

## Abbreviations

CNS: Central nervous system
COREQ: Consolidated criteria for reporting qualitative research
DIPEx: Database of individual patients’ experiences of illness
DMT: Disease-modifying therapy
ICF: International classification of functioning, disability, and health
INIMS: Institute of Neuroimmunology and Multiple Sclerosis
MS: Multiple sclerosis
PDDS: Patient determined disease steps
PExMS: Patients’ experiences with multiple sclerosis
PPMS: Primary-progressive multiple sclerosis
pwMS: Persons with multiple sclerosis
RRMS: Relapsing–remitting multiple sclerosis
SMART: Specific, measurable, achievable, realistic/relevant and timed
SPMS: Secondary-progressive multiple sclerosis
WHO: World health organization
